# Reproductive Ecology of Male and Female Strobili and Mating System in Two Different Populations of *Pinus roxburghii*

**DOI:** 10.1100/2012/271389

**Published:** 2012-04-30

**Authors:** Chandra Mohan Sharma, Vinod Prasad Khanduri, Sunil Kumar Ghildiyal

**Affiliations:** ^1^Department of Botany, HNB, Garhwal University, Srinagar Garhwal 246 174, India; ^2^Department of Forestry, Mizoram University, Aizawl 796 001, India

## Abstract

We studied several flowering traits, namely, male-female cone phenology, male-female cone production per tree, mating system, sex ratio, air-borne pollen grains and pollen migration, over four successive years in two different natural populations of *P. roxburghii* from Garhwal Himalaya, India. Assessment of each trait mentioned except pollen dispersion was done by selecting five representative trees randomly in each population. The pollen migration was studied on naturally isolated source trees. The pollen trapping was done in all directions up to 2.5 km. The average reproductive period in *P. roxburghii* was 36 days with 3–5 days protandry. There were significant year and population effects for male and female cone output and pollen grains production per tree. In mass production year (1999), an average production of pollen cone per tree was estimated as 42.44 ± 8.32 × 10^3^ at lower altitude and 28.1 ± 0.89 × 10^3^ at higher altitude. The controlled pollination results in high level of outcrossing with 90% seed setting. We conclude that the high male-female ratio and tremendous pollen production capacity in *P. roxburghii* indicate high male competition among trees within populations. The isolation strip of 600 m is considered minimal for the management of seed orchard.

## 1. Introduction

The *Pinus roxburghii* Sargent in Silva of North America in September 1897 (commonly known as Chir-pine or Himalayan Long needle pine) is native to Himalaya, which occurs naturally between 450 and 2300 m asl. It covers wide areas as pure forests and sometimes also found mixed with other coniferous and broad-leaved species. It is a fire resistant and strong light demander tree species which has excellent regenerative potential. It is an anemophilous and monoecious species, pollination takes place in the spring, and fertilization occurs in the next spring; however the mature cones disperse the seeds in third spring after pollination. Thus the time elapsed between pollination and seed maturity is 24 to 26 months [1]. The rotation age of *Pinus roxburghii *in Himalaya is 120 years. At this age the average stem wood yield is 835.5 ± 32.4 m^3^/ha, whereas total wood yield is 1011.5 ± 54.56 m^3^/ha. The average diameter of the trees at rotation age is 72.6 ± 4.53 cm and average height is 42.8 ± 1.12 m [2]. The sexual maturation age ranges from 12 to14 years for pollen cone and 17 to 20 years for seed cone production. At higher elevations the *Pinus roxburghii* prefers hotter slopes and drier spurs, but at lower altitudes cool northerly slopes. It is absent from the areas where full force of monsoon is not felt [3].

Tree-improvement programmes for chir pine (*Pinus roxburghii*) are developing throughout its range. So far, two Seed orchards of this species have been established in the Western Himalayan region, that is, one by Forest Research Institute (FRI) in Uttarkashi district of Uttarakhand state, and other by Himalayan Forest Research Institute (HFRI), in Shimla district of Himachal Pradesh state (India), both are still in developing stage, not yet attained the sexual maturity age, and others are planning to produce regular crops of high-quality seeds for reforestation. Chir pine is slower to produce seed cones with intermittent production than pollen cones regularly every year that would be difficult to seed orchard managers to promote sustainable cone and increase seed production. The abundance of pollen cone per individual tree represents the quantity of male gamete in the population which follows the pattern by which gametes are transmitted from the male to the female. This pattern is referred as the mating system of a species. The mating system is a functional relationship that allows us to predict how the gametes from the mature population combine to form the viable zygotic population. High production of pollen cones alongwith blooming synchrony and asynchrony of cones creates a theoretical platform for describing the mating system [4]. The timing of flowering and synchronization among individuals influence the quantity and quality of seed production [5]. The diurnal anthesis rate in chir pine enhances the possibility of pollination success, as the pollen grains dislodge from the dehisced anther if there is some disturbance and in nature wind is the only disturbing agency to emit the chir pine pollen grains into the atmosphere. The wind velocity increases as the day progresses and attain maximum velocity from 1300 to 1700 h of the day [1]. Furthermore, the anthesis cycle of a pollen cone varies from lower to higher elevation with 2.5 and 6.0 days respectively, which is highly influenced by air temperature and atmospheric humidity, moreover not affected by light intensity [6]. The best temperature range to ascertain quickest anthesis is 25–28°C [6]. This finding is very important to create temporal isolation to the desired families in the seed orchard for further advancement of tree improvement in this species.

The quantity of pollen produced by an individual tree has the tremendous role for successful seed setting in *Pinus* where the pollen reaches to the receptive strobili via wind and the physical structure and bract scale of female strobili do not allow the pollen grains easy to reach the surface of integument of the ovule. Each bract scale contains two ovules and there is 55–75 bract scale in a female cone which require ample amount of pollen grains in every direction of the female cone for successful seed set. This is only possible if huge quantities of pollen are produced [7]. *Pinus roxburghii* produces high amount of pollen per cone and per tree as compared to other *Pinus* species; henceforth the seed setting following open pollination is also high, that is, 90% with high seed germination of 92 percent [8]. The levels of pollen production influence the efficiency of wind pollination due to the poor pollen flow and concentration of airborne pollen that ultimately hinder both ovule fertilization and seed production. Therefore, the estimation of total pollen production is important, as production of seeds depends on the production of pollen [9, 10].

Despite the widespread focus on pines in genetic and tree-improvement programmes, little information has been found towards the number of pollen cones per tree, pollen production per cone, and female cone production. Collecting data yearly on the seed-cone and pollen crops is very important in order to quantify the balance in reproductive effort [11]. This will have an impact on the genetic balance in seed orchard seed crops. Genetic variation exists among trees in nature should be known in order to implement efficient tree improvement programmes, with respect to sustainability. Data on the reproductive mechanism of population of tree species are indispensable for effective genetic conservation and as working tools to our understanding of evolutionary mechanisms.

Taking into consideration all above facts in mind, the objectives of this study were to: (1) examine male and female cone phenology and its role in the mating system, (2) estimate the level of variability in the production of male and female cone among years, between populations and within a population, and (3) estimate the effective pollen migration distances from isolated source trees. The findings of this study would be valuable to forest managers, breeders and those working on the population structure of conifers, because the reproductive system of this species is exclusively sexual, which has not been unraveled so far in this important resin-timber resource of Himalayan region.

## 2. Materials and Methods

### 2.1. Study Sites

**Figure 1:**
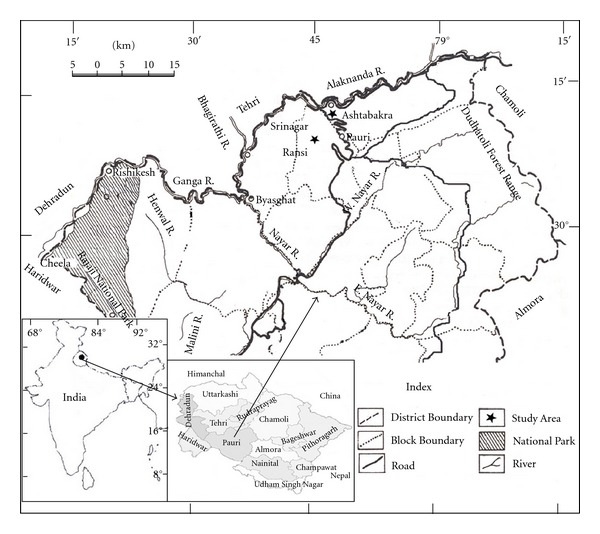
Location map of the study area.

This study was conducted during January to March each year for four successive years, that is, 1998, 1999, 2000, and 2001 in two different natural chir-pine forests of district Pauri Garhwal (latitude 29° 20′ to 30° 15′ N and longitude 78° 10′ to 79° 20′ E). Both the forests were growing at two different elevations: (i) lower elevation (Ashtabakra: 900 m. asl.) and (ii) Higher elevation (Ransi: 1900 m. asl.) in the central part of Western Himalaya (Figure 1 and Table 1). More details of the study area are presented in Table 1.

### 2.2. Assessment of Development of Cones

Five trees from the natural population of chir-pine forest were selected randomly at each altitude and marked by yellow paint. These trees were considered as the representative individuals for all studied variables and the same trees were used for 4-successive years sampling. The stand density at lower altitude was 190 individual per ha, which at higher altitudes was 160 individuals per hectare (excluding other species). The spacing between the trees was 7 × 7 m at lower and 8 × 8 m at higher altitudes, respectively, which clearly indicates that the light condition was uniform in both stands. The forests on both elevations were mature and the pattern of distribution of trees over the populations was regular with proper spacing. The size of trees was approximately identical within each altitude, as the average diameter and height of the stand was 63.4 ± 1.16 cm and 31.0 ± 0.50 m, respectively, at lower altitude and 64.6 ± 1.34 cm and 31.8 ± 0.58 m, respectively, at higher altitude. The stand density was estimated by laying five sample plots of 0.1 ha each on each elevation and individuals encountering within the sample plots were measured for DBH (diameter at breast height) and height. Three stages were monitored for the development of cones which include (i) date of cone initiation (the time when pollen cone and seed cone start flushing on the shoot apices), (ii) pollen cone maturation (the stage just before anthesis when pollen grains were fully developed), and (iii) blooming of pollen cones and time of seed cone receptivity (The stage when anther dehiscence took place and pollen grains were ready to flow into the ambient air and female/seed cone receptivity started). To observe these developmental stages of pollen cone and seed cone, five branches containing pollen and seed cone buds were chosen randomly on the whole crown, covering all geographic directions of five different trees in each location and were monitored at one week's interval between mid-January and Mid-February, and two times per week between mid-February and the end of March, until pollination ended on both altitudes. The daily rates of anthesis were recorded on ten randomly chosen pollen cones per branch and fifty pollen cones per tree on the five selected individuals in each altitudes, till all the strobili in a pollen cone were opened. The pollen cones were examined after every two-hour interval for the entire day length (0600 to 1800 h) till the completion of anthesis cycle (opening of all strobili in a cone and subsequently for all selected cones per tree, and the average value was considered as the time taken for anthesis, referred to as anthesis cycle). The counts on opened strobili were made within a pollen cone and on all fifty pollen cones of each tree and subsequently for five trees at each elevation, by scoring and removing method, to avoid duplication. Simultaneously, the timing and rate of microsporangium dehiscence on ten representative strobili per cone and ten cones per tree were recorded with the help of a hand lens (×20) by counting the dehisced microsporangia in a similar way. The levels of prevailing air temperature and relative air humidity were also recorded close to the pollen cone during each observation with the help of a thermohygrometer.

### 2.3. Pollen Production and Number of Pollen and Ovulate Cones

For the assessment of production variables, first the main branches were counted, and then a sample of five branches on the same trees which have been selected earlier was chosen randomly throughout the crown, taking into consideration the smaller branch on top of the crown and medium and large branches on middle and lower parts of the crown, respectively, in all directions (southern and northern sides of the crown), and all pollen cones were counted. The results were then multiplied by the total number of branches to achieve an estimate of total pollen cone production per tree. Further, twenty pollen cones, scattered throughout the tree were harvested, and the number of pollen strobili per pollen cone was counted for each tree. From each pollen cone, pollen strobili were chosen from the lower, middle and upper parts, and the numbers of microsporangia were counted manually. The assessment of pollen grains was done on five microsporangia from different pollen strobili of each tree as per the method suggested by Molina et al. [12]. In order to estimate total production of pollen grains per tree, first the total number of microsporangia per tree was calculated by multiplying the total number of pollen cones by the average number of pollen strobili per pollen cone, and then by the average number of microsporangia per pollen strobilus. The result was then multiplied by the average number of pollen grains produced per microsporangium. This process was repeated every year for estimation of total pollen production.

The counting of ovulate strobili production was same as described for pollen strobili production. The ovulate cone production is very low in *P. roxburghii *and the census is easy with the help of identification by colour marker. The female strobili grow on the top of unflushed buds (red colour) of 5 to 8 cm long. Flushing of needles from these buds starts after completion of flowering. However, the counting was done very carefully. First, five sample trees were chosen, and then the total number of branches that produced ovulate strobili per tree was counted. Five branches of each tree were chosen randomly for counting the ovulate strobili per branch. The results were multiplied by the total number of branches to achieve an estimate of total ovulate strobili production per tree.

#### 2.3.1. Sampling of Air Borne Pollen Grains and Pollen Adhesion on Ovulate Cone Strobili

The presence of pollen in the atmosphere was observed by taking pollen bearing air samples on five selected source trees in a stand. The pollen capturing was recorded on 15 jelly-coated microscopic slides set at 0600 h of the day and five slides were removed at every 2-hour (0800, 1000, 1200, 1400, 1600, and 1800 h), 6 hour (1200 and 1800 h), and 12-hour (1800 h) intervals up to 5-days and was repeated in each of the four-year study. The slides which were taken out after every 2-hour and 6-hour intervals were replaced by new slides. These slides were mounted vertically on iron rods and were placed perpendicular to the direction of the prevailing wind and all around the source tree at a distance according to the spread of crown. The rod was mounted on the trees at heights corresponding to the height of the pollen cones. The number of pollen grains per slide was counted under the binocular microscope on an area of one cm^2^ (1 × 1 cm). The wind speed was recorded by a digital anemometer. The deposition of pollen grains on ovulate cone strobili were assessed by bagging hundreds of strobili on five different trees at the initial stage of development before receptivity. The ovulate strobili were then exposed to wind during receptivity period in batches at every 2 h intervals for the entire day length up to three days. The strobili were removed after every 2 h interval from the source tree and assessed for the number of pollen grains deposited on scalebract complexes, sensu Ornduff [13].

#### 2.3.2. Pollination

A study was set up in the year 1999 to determine the cone and seed setting after controlled pollination on two altitudes. Ovulate cones on some easily approachable trees at both altitudes were enclosed in pollination bags before receptivity and the experiment was performed for geitonogamous self-pollination, cross pollination, and open pollination. Pollen grains or pollen pool for self-pollination was used from the same individual that has been selected for controlled crosses, whereas, for cross-pollination, pollen grains were collected from five different trees mixed and blown to the ovulate strobili. Pollen grains blowing into the pollination bags were done at the time of receptivity through hypodermic syringe.

#### 2.3.3. Sex Ratio

Sex ratio of flower on a single individual tree level [14] was done in this study. It was determined by dividing the estimate of the number of pollen cones per tree by the number of ovulate strobili per tree.

#### 2.3.4. Observation on the Pollen Dispersion

Two ideal chir pine pollen source trees were selected, one each at two different altitudes. In both cases the trees were naturally isolated from other pollen sources (*Pinus roxburghii* trees) by a radius of more than 1.5 km. Despite the distance of 1.5 Km, the geographical barriers (e.g., (i) hill tops, (ii) denuded hill slopes, (iii) rocky outcrops, and (iv) forests of other species) were also present for isolation of the source trees on both altitudes. Nevertheless, the effective pollen migration of chir pine from whole stand was observed up to 1.28 km from the source [15]. Therefore, there was no possibility of errors in terms of pollen contamination from other source trees. Pollen samples at the lower altitude were collected, (i) up to 0.64 km (i.e., at distances, 0, 05, 10, 20, 40, 80, 160, 320, and 640 m) horizontally (with an average slope of ±5°) opposite to the prevalent wind direction, (ii) up to 0.32 km, in the vertically uphill direction (average slope 28°), and (iii) up to 2.5 km, in the vertically downhill direction (average slope 37°) parallel to the average wind direction. On the other hand at the higher altitude, pollen sampling was done (i) up to 2.0 km (0, 05, 10, 20, 40, 80, 160, 320, 640, 1280, and 2000 m) horizontally (average slope ±3°) towards the prevailing wind direction, (ii) up to 0.32 km, in the vertically uphill direction (average slope 57°), and (iii) up to 2.5 km, in the vertically downhill direction (average slope 42°) to give adequate estimates of frequencies near the source. These distances in different directions were accessible and were free from any geographical barrier. Samples were taken over four days at each altitude and all repeated during each of the four years (i.e., 1998, 1999, 2000 and 2001). Ordinary microscopic slides, covered with a thin coat of petroleum jelly (Vaseline), were used as pollen traps. The slides were fastened in horizontal position on a 2.5 m long staff established on the ground, which were unprotected and were exposed in the open air. The slides were set out between 4 and 5 pm daily and collected after an interval of 24-hr. Pollen counts were made directly from the exposed glass slides, under the binocular microscope. The area counted per slide was fixed that is, 1 cm^2^. The experiment was replicated for four successive years; however, the basic design of the experiment on pollen dispersion study was same as reported earlier by the authors [15].

#### 2.3.5. Statistical Analysis

Analysis of variance was used to test for year, population, and tree as fixed effects. The cone and pollen production counts were log transformed in order to improve normality of residuals and to reduce heteroscedasticity [16], which was analysed in both populations over 4 years of observations. ANOVA was performed using SPSS package. Pearson correlation was used to assess the relationship between climatic variables (temperature and relative humidity) versus microsporangium dehiscence as well as the first date of flowering versus the length of flowering period at lower and higher altitudes.

## 3. Results

### 3.1. Pollen Cone Development, Anthesis, and Microsporangium Dehiscence

Observations on the development of pollen cones for four years have revealed that the initiation of pollen cone buds started from lower to higher altitudes (at lower altitude the initiation was recorded between 10th and 20th January, whereas at higher altitude between 30th January and 2nd February during the years 1998 to 2001). The pollen cone blooming was varied between 18th February and 26th February at lower altitude and 4th March and 13th March at higher altitude. However, the variation in timing of cone initiation, maturation, and blooming among individuals within the populations in one year was also observed, which laid out the foundation for the conceptual description of asyncronization in flowering. A seed cones of *P. roxburghii* produce 84.6 ± 14.16 mean number of seeds per cone that varies considerably and depends on the position of the cone in the crown. The seed cones that grow on upper part of the crown produce 90.2 ± 3.04 seeds per cone, which on the middle part of the crown contains 80.4 ± 6.14 seeds per cone, whereas, the cones appear at the lower parts of the crown produce only 66.0 ± 3.41 seeds per cone [17].

**Figure 2:**
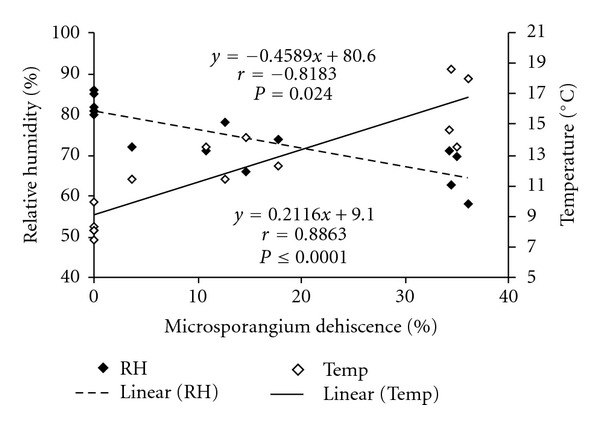
Relationship between microsporangium dehiscence versus temperature and relative humidity.

Anthesis occurred between 06:00 and 18:00 hr of the day, which was dependent entirely on the levels of air temperature and atmospheric humidity. The anthesis cycle varied considerably at lower and higher altitudes, because a pollen cone took 2 to 3 days to complete the anthesis cycle at lower altitude and 5 to 7 days at higher altitude (Table 2), due to subsequent decrease in air temperature and increase in atmospheric humidity at higher altitude. The peak period of anthesis was recorded between 12:00 h and 14:00 h of the day at both altitudes, because of occurrence of maximum temperature and minimum atmospheric humidity during this period. The microsporangium dehiscence has followed the same pattern as anthesis, with a slight variation that it peaked between 10:00 h and 16:00 h of the day. Although the rate of anthesis in pollen-cone is primarily determined by magnitude of available temperature but microsporangium dehiscence and pollen release are also determined by humidity. A light rain can delay dehiscence by one day or more [18]. The pattern of microsporangium dehiscence was lateral at strobili level, that is, from broader apical to narrower basal end, with two longitudinal lines gradually widening towards the basal end. Significant positive and negative correlations were also observed between microsporangium dehiscence with temperature and relative humidity, respectively (Figure 2).

### 3.2. Sexual Perspectives, That Is, Pollen Quantity and Sex Ratio

*P. roxburghii *exhibits remarkable variation in the degree of differentiation of male and female gametes (Table 3). The production of pollen cone per tree varied from 18.0 × 10^3^ to 60.7 × 10^3^ at lower altitudes and 17.0 × 10^3^ to 31.6 × 10^3^ at higher altitudes; however, the ovulate cone production per tree ranged from 18 to 221 at lower altitudes and 10 to 154 at higher altitudes, which also varied considerably from one year to the next. The ANOVA results for the production of pollen per tree revealed significant variation among years, between altitudes, and between year and altitudes interactions. Similar significant differences were also observed among years, between altitudes and between year and altitudes interactions for other variables that is, pollen strobili per tree and microsporangia per tree. However, the variation in the production of microsporangia per strobilus and pollen grains per microsporangium among years is not significant but less significant between altitudes (Table 5). The results of ANOVA finally revealed that there is temporal and spatial variations in the production variables in *P. roxburghii*, which clearly suggest that the climatic conditions play a significant role in this variation.

### 3.3. Pollination and Dispersion

Successful pollination is usually measured by pollen flight or by pollen attached to the micropylar arms of the ovulate strobili. Diurnal pattern of pollen concentrations in the air was observed in *P. roxburghii*, which depends on the extent of air movement and/or the magnitude of atmospheric turbulence, that vary according to the variation in air temperature throughout the day. Within a day length (between 06:00 and 18:00 h) the highest frequency was observed between 12:00 to 14:00 h and 14:00 h to 16:00 h (Table 4), during which peak pollen deposition on scale-bract complexes was also noticed, therefore this time is considered to be the best time for pollination within a day. Ovulate cone initiation was noticed from February 18th to February 24th at lower altitude, whereas on higher altitudes it was noticed from March 4th to March 10th in years 1998 to 2001. The time of receptivity of ovulate cones was recorded between 23rd February and 2nd march at lower altitudes, whereas at higher altitudes, it was during 9th to 17th March.

Dispersion of pollen grains in *P. roxburghii* was recorded during the peak flowering periods on both higher and lower altitudes. The observations revealed that pollen frequencies near the source tree were highest in all uphill, downhill, and horizontal directions (Figure 4). Effective dispersion of pollen grains was recorded, up to 320 m in uphill direction, and up to 640 m in horizontal and downhill directions, and the mean pollen frequencies relative to the source frequencies at these distances were 0.69–3.25%, 0.56–4.33%, and 3.43–7.21%, respectively. In the uphill and horizontal directions pollen grains traveled only up to 320 and 1280 m (Figure 4). However, in the downhill direction pollen grains could migrate up to 2520 m but the pollen frequency relative to the source frequency at this distance was very low, that is, 0.2%. The trends were similar in all the four studied years and were also at par with our previous observations [15].

**Figure 3:**
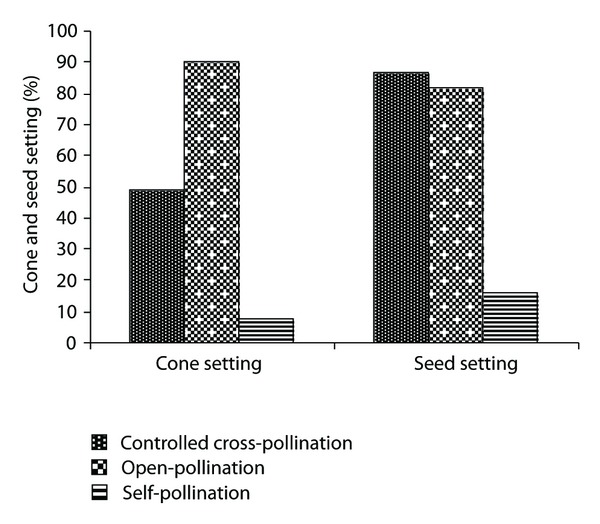
Cone and seed setting after controlled pollination.

The observations on controlled pollination experiments revealed that 85 to 90% of the cones survived up to maturity in open-pollinated condition, whereas 46 to 52% cone setting was observed in controlled cross-pollinated condition. The maximum cone loss was observed soon after pollination, which may be because insufficient numbers of ovules were being pollinated in controlled pollination. The cone and seed setting in self-pollinated condition was very low (Figure 3). 

## 4. Discussion 

The variation in flowering phenology (the study of the events occurring periodically as influenced by the environment, especially temperature changes driven by weather and climate [19]) was observed from year to year within each of the population studied at lower and higher altitudes [19]. There is a difference in pollen release (movement of pollen during microsporangium dehiscence) and ovulate cone opening (referred as receptivity of female strobili in conifers). The later starts 3–5 days after pollen release, clearly suggesting the protandrous dichogamy in *P. roxburghii*, which has important consequences for promoting outcrossing in this species, as there is asynchrony in blooming of pollen cones within the population. Trees that flower early tend to have shorter flowering period as a result the first flowering date was significantly negatively correlated with the length of flowering period (*r* = −0.568, *P* < 0.001) at higher altitude. However, the relationship was weaker (*r* = −0.384, *P* < 0.462) at lower altitude. In *Pinus contorta,* the phenology varies by about two weeks from year to year depending on spring weather [20]. 

It is a matter of further research that despite earlier maturity of male strobili the pollination and fertilization rates in terms of seed setting and seed germination are considerably higher in *Pinus roxburghii* than other pine species. This is due to the fact that the pollen emission started earlier than female receptivity and lasted for longer period of time, which promote the outcrossing level in the population. The overall reproductive period was 36 days, which is comparable to that found in other conifers [21, 22] and broadleaved, like teak [23]. 

The receptivity of ovulate cone in *Pinus roxburghii *varies form 3 to 5 days. The period of receptivity of ovulate cone strobili observed in *Pinus contorta* varied from five to seven days with the maximum receptivity occurring for two to four days [20]; in *Pinus sylvestris *it lasted from 3 to 10 days [24]. In other coniferous species the receptive period of ovulate cone strobili varied from 2 to 7 days in Norway spruce [25], 5 to 12 days in Douglas fir [26], 6 to 8 days in Sitka spruce [27], 12 days in Black spruce [28], and 10 days in White spruce [29]. Ovulate cone strobili in *P. roxburghii *harbour two ovule primordia on the basal adaxial surface of the fertile scales. Some scales in the basal portion of the cone lacked ovules or formed only rudimentary ovules. Pollination success is a measure of the amount of pollen deposition on ovulate strobili, which usually correlates well with the amount of pollen in the air [7], as measured by taking pollen air samples. The maximum concentration at both lower and higher altitudes was observed from 13:00 h to 16:00 h of the day (Table 4). 

Controlled pollination studies revealed that majority of cross-pollinated cones developed normally till seed maturity, whereas the self-pollinated cones developed normally only up to 12 months from pollination mostly and thereafter they became dry. Seed setting in cross-pollinated cones was slightly higher as compared to open-pollinated cones and difference between cross and open pollinated seed setting is significant (*P* < 0.01). However, for other conifers the cross- and self-pollinated studies are well documented, which have shown that self-pollination in conifers in general [30] and particularly in *Pinus* [31] results in abortion of embryo and megagametophyte, within a few weeks after fertilization [32, 33], and the aborted seeds are called as the “empty seeds.” The interracial hybridization is possible in *Pinus *roxburghii and about 40 to 55% cone setting and 72 to 87% seed setting were recorded after controlled crosses between different provenances [34]. Outcrossing rates in conifers have been estimated high at the population level [35–41]. 

Flowering and pollen production under different environmental conditions are highly variable in *P. roxburghii*. Pollen production levels within the same climatic region are strongly same and could be varied due to the effect of plant density, size class, vigour, site, and meteorological phenomenon such as sunshine, temperature, wind direction, velocity, and turbulence [42–44]. Therefore, the altitude plays the crucial role for the production of reproductive variables in *P. roxburghii*, which at the lower altitude produce significantly more pollen cones every year, moreover temporal variation is similar on both altitudes with the pattern of biannual production. The interannual differences in cone production have also been reported for *Cedrus atlantica* natural population [45]. The magnitude of variation in the production of pollen cones in *Pinus roxburghii* from one year to the next is high at lower altitude as compared to higher altitude. The difference between high and low production years at lower and higher altitude is 28% and 21% (between 1998 and 1999) and 39% and 24% (between 2000 and 2001), respectively. Lower altitude produced 25% more pollen cones as a whole for four studied years. Positive significant relationship between climate and cone production was recorded for *Abies balsameia *[46] and *Picea mariana *[47]. This variation could minimize the pollen dispersion rate, which ultimately affects the mating system and the chances of variability are more between the population distributed spatially at narrower distance at the upper range of distribution of this species. Furthermore, the results also suggest that this variation is due to different genetic composition on both the locations, which would have been formed due to the effect of altitude and adapted to local environment. The effect of altitude would be minimum between 1500 and 1600 m asl (intermediate altitude), which is considered to be the best range for the establishment of SSOs. Alternately, the differences in production variables are genotypic in addition to the physical, biological, and environmental factors. Also both locations have been marked as two different provenances or geographic races by Ghildiyal et al. [8]. The production of ovulate cone strobili shows that masting behaviour, the intermittent production of large crops of flowers or seeds by a plant population, is a common feature among many plant species in boreal and temperate zones [48]. The year of mast production was 1999 at both altitudes. Many theories have been presented to explain the ultimate and proximate causes of masting [49]. In *Pinus sylvestris*, it was noticed that the possibility of seed production is predicted on the basis of weather factors, providing that the energy loss in trees caused by prolific flowering and seeding does not affect seed production during the following year. In *Picea abies*, a good flowering year is usually followed by one or several poor flowering years [50]. In birch species (*Betula pendula* and *B. pubescens*), the masting is regulated by weather factors together with the system of resource allocation among years [51]. In the Himalayan region of India, *Cedrus deodara* also contributes masting pattern in the production of pollen cone [52]. 

**Figure 4:**
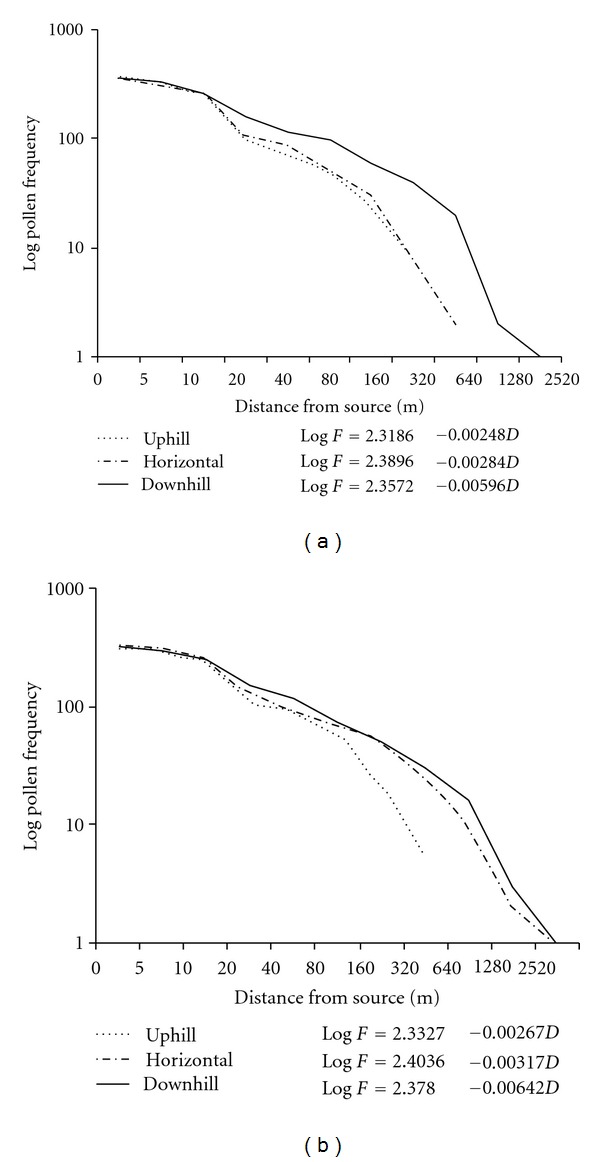
Pollen dispersion at different distances from source tree at lower and higher altitudes.

The results pertaining to pollen dispersion in *P. roxburghii* indicate that the concentration of pollen drops off rapidly with distance and the highest densities occurred within 50 m from the source, which is well supported by several other studies [15, 53–56]. The pollen flight distances ultimately lead to suggest that an isolation strip of 600 m is considered minimal for the management of *P. roxburghii *seed orchard. The pollen output by an individual tree of *P. roxburghii *is tremendous and quantity of pollen transported over long distances is small. However, considerable amount of pollen could travel over long distances when whole stands are taken as pollen source [15, 57]. The overall results indicate that the frequency of air-borne pollen declines rapidly as the distance from the source increases (Figure 4). This rapid decline is of great practical value in the evolutionary biology of this species. As a consequence development of new races in nature would be more pronounced at higher altitudes where the forests of *Quercus leucotrichophora* are being gradually encroached by chir-pine forests [58]. The chir pine has a wider adaptability in subtropical to middle temperate zones; consequently the species may suppress or replace many other forest forming species in near future including *Quercus leucotrichophora* [58]. 

The reported pollen frequency, for example, in *Pinus edulis* at 90 m was 1 percent relative to the pollen source [53], and in *Pinus elliottii* it was 2 to 5% at 150 m from the source [54]. DNA markers also have been used to investigate level of pollen contamination into conifer seed orchard [59]. For example, the isolation distance recorded for *Pinus glauca* seed orchard is 1000 m [60] and for *Pseudotsuga menziesii* 500 m [61]. *Pinus taeda *[62] and *Pinus sylvestris *[63] seed orchards were separated by 200 m and 2000 m, respectively, from the nearest stands of the same species. The pollen dispersion data of a species are essential for the estimation of the extent to which isolation is important for race formation, and in forest stands, it is of fundamental importance particularly in forest management programmes, where tree harvest and the practice of leaving scattered seed trees are to be decided. 

Choosing isolated trees as a model for pollen dispersal study has the advantages, namely, (i) possibility to escape the determination of equivocal paternity, which is more often related with the studies of pollen migration observed within larger and denser tree populations [64], and (ii) the dispersal distance of 600 m from an isolated tree represents a vital level of long-distance immigration, in evolutionary terms [65]. The long distance movement of pollen in *Pinus roxburghii *is also confirmed by the similar results observed for *Pinus flexilis *[66] and *Pinus sylvestris *[67]. Furthermore, the dynamics of airborne pollen in forested sites is influenced by wind turbulences linked with vertical and horizontal structures [68] and the low-density populations enhance the pollen flow [69]. The pollen flow results of this study confirm that pollen scattering regulates on a wide scale within and among pine populations. The production of pollen grains per tree and density of individuals are factors that may strongly determine the extent of effective pollen dispersal and the mating system pattern within stands and provide the concern in near future for evolutionary and conservation studies of *P. roxburghii*. 

## 5. Conclusions 

The results of this study show that the phenology of male and female strobili and their production in *P. roxburghii *varied from year to year and altitudinaly, thus, the climatic conditions play crucial role. The pollination system of this species is exclusively out-crossing and the cone and seed setting from self pollination is very low which leads to high seed setting (90%) and high seed germination (92%). The best time of the day for maximum pollination is between 12:00 and 16:00 h, which was also confirmed by pollen adhesion pattern onto the megasporophylls and verified by the trapping of pollens on jelly-coated slides exposed to the air. The pollen-ovule ratio entails greater variance in reproductive effort of males as compared to females. The high male-female ratio and tremendous pollen production capacity in *P. roxburghii *indicate high male competition among trees within populations for successful out-crossing and maximum seed set through sufficient pollen grains reaching each megasporophylls, which addresses ultimately the evolutionary cause of this pattern. The results of the pollen dispersion suggest that an isolation strip of 600 m is minimal for the management of *P. roxburghii *seed orchard; however, the best altitude for its establishment would be 1500–1600 m asl, where the environment for its growth will be most suitable. Furthermore, the entire study leads to conclude that the mating patterns of *P. roxburghii* at the stand and population levels have the influence of the natural factors, that is, size and density of tree population, phenological patterns, flowering synchrony, male and female reproductive effort, and mode of pollination. 

## Figures and Tables

**Table 1:** Specifications of the sites.

Population/site	Altitude	Latitude	Longitude	Mean annual temperature (°C)	Total rainfall (mm)	Density (stems/ha)	Other species
Ashtabakra	900 m. asl	30° 13′	78° 48′	22.1 ± 1.76	1495.9	190	None in tree layer
Ransi	1900 m. asl	30° 09′	78° 48′	14.63 ± 2.06	1635.00	250	*Quercus leucotrichophora, Rhododendron arboreum, Pinus wallichiana*, *Cupressus torulosa *

**Table 2:** Reproductive phenology of *Pinus roxburghii. *

Observed variables	Lower altitude (900 m asl)				Higher altitude (1900 m asl)			
	1998	1999	2000	2001	1998	1999	2000	2001
Date of Pollen cone bud initiation	January 16th	January 13th	January 20th	January 10th	February 2nd	January 30th	February 6th	January 30th
Date of Pollen cone maturation	February 12th	February 10th	February 14th	February 8th	February 27th	February 24th	February 27th	February 26th
Date of Pollen cone blooming	February 23rd	February 18th	February 26th	February 21st	March 13th	March 04th	March 10th	March 6th
Anthesis cycle of pollen cone	3 days	2 days	3 days	2.5 days	7 days	5 days	6 days	6 days
Peak period of anthesis within a day	1200 and 1400 h	1200 and 1400 h	1200 and 1400 h	1200 and 1400 h	1200 and 1400 h	1200 and 1400 h	1200 and 1400 h	1200 and 1400 h
Min.-Max. temperature (°C) within the study period during anthesis	7.3−18.2	8.5–19.3	7.0–18.5	7.6–20.0	7.2–14.3	7.8–17.2	6.6–17.0	7.4–18.0
Min.-Max. R. H. (%) within the study period during anthesis	64–82	62–80	60–80	60–80	70–86	68–84	70–85	65–85
Min.-Max. wind speed (m/s) within the study period during pollen grains in the air were sampled	0.8–3.8	0.6–2.4	0.4–2.8	0.8–3.0	0.8–3.0	0.6–3.5	0.6–3.2	0.8–3.5
Date of ovulate cone initiation	February 22nd	February 18th	February 24th	February 21st	March 10th	March 04th	March 08th	March 05th
Ovulate cone receptivity period	4 days (Feb. 27th–March 2nd)	3 days (Feb. 23rd–Feb. 25th)	3 days (Feb. 27th–Mar. 1st)	3 days (Feb. 26th– Feb. 28th)	5 days (Mar 13th– March 17th)	5 days (Mar. 09th– March 13th)	4 days (Mar. 12th–March 15th)	4 days (Mar. 10th–March 13th)

**Table 3:** Production potential of reproductive organs in *P. roxburghii. *

Observed variables	Lower altitude (900 m asl)				Higher altitude (1900 m asl)			
	1998	1999	2000	2001	1998	1999	2000	2001
Pollen cone production per tree (×10^3^)	28.3 ± 1.56	42.44 ± 8.32	22.6 ± 1.72	33.60 ± 6.78	21.9 ± 0.34	28.1 ± 0.89	20.3 ± 1.22	24.48 ± 1.78
Ovulate cone production per tree	101.0 ± 2.13	210.4 ± 3.54	27.0 ± 3.49	47.6 ± 5.52	57.4 ± 0.87	143 ± 3.76	17.2 ± 2.92	33.9 ± 4.7
Pollen grains production per cone (×10^6^)	71.9 ± 4.08	77.22 ± 9.76	68.58 ± 3.62	72.12 ± 8.2	66.8 ± 2.9	68.1 ± 3.25	66.3 ± 2.19	67.2 ± 3.58
Pollen grains production per tree (× 10^11^)	22.1 ± 1.37	27.66 ± 3.15	22.04 ± 1.41	23.6 ± 3.29	15.4 ± 1.23	18.3 ± 0.76	13.9 ± 1.48	16.2 ± 1.25
Ovulate cone production per 100 male cone	0.36 ± 0.01	0.65 ± 0.003	0.158 ± 0.015	0.216 ± 0.02	0.26 ± 0	0.57 ± 0.01	0.14 ± 0.01	0.18 ± 0.02
Pollen-ovule ratio/individual	25.1 ± 1.59	13.16 ± 3.98	85.48 ± 0.89	53.4 ± 4.95	26.1 ± 0.99	12 ± 2.08	74.6 ± 2.2	53.5 ± 2.52

**Table 4:** Temporal and spatial variation in frequency of air borne pollen in *P. roxburghii. *

Observed variables	Lower altitude (900 m asl)				Higher altitude (1900 m asl)			
	1998	1999	2000	2001	1998	1999	2000	2001
Pollen concentrations in the air between 1200 and 1600 h of the day (pollen/cm^2^)	119 ± 9.84	160 ± 12.19	140 ± 8.34	180 ± 10.14	98 ± 4.34	215 ± 18.79	142 ± 9.12	186 ± 16.34
Pollen deposition per scale-bract complex in the ovulate cone between 1200 and 1600 h of the day (pollen/bract scale)	17 ± 1.16	36 ± 4.73	26 ± 2.12	32 ± 6.16	22 ± 2.57	29 ± 5.12	42 ± 2.84	52 ± 8.12

**Table 5:** ANOVA of the effect of year and altitude on number of Pollen strobili, microsporangia, and pollen grains per tree in *P. roxburghii. *

Response variable and source	1				2				3				4				5			
	a	b	c	d	a	b	c	d	a	b	c	d	a	b	c	d	a	b	c	d
df	3	1	3	680	3	1	3	487	3	1	3	741	3	1	3	782	3	1	3	467
*P*	0.000	0.0014	0.0263		0.2442	0.0122	0.2876		<0.0001	0.0005	0.0292		0.0001	0.0002	0.0146		0.2246	0.0112	0.2672	

1:  Number of pollen strobili per tree (*R* ^2^ = 0.692), 2:  Number of microsporangia per strobilus (*R* ^2^ = 0.568), 3:  Number of microsporangia per tree (*R* ^2^ = 0.856), 4: Number of pollen grains per tree (*R* ^2^ = 0.612), 5:  Number pollen grains per microsporophyll (*R* ^2^ = 0.468). a: Year, b: Altitude. c: Year × altitude, d: Error.
